# Inhibition of the Otub1/c-Maf axis by the herbal acevaltrate induces myeloma cell apoptosis

**DOI:** 10.1186/s12964-020-00676-w

**Published:** 2021-02-24

**Authors:** Tong Sun, Yujia Xu, Zhuan Xu, Biyin Cao, Zubin Zhang, Qi Wang, Yan Kong, Xinliang Mao

**Affiliations:** 1 Institute of Clinical Pharmacology, Science and Technology Innovation Center, Guangzhou University of Chinese Medicine, Guangzhou, 510405 People’s Republic of China; 2 Department of Neurology, the First Affiliated Hospital of Soochow University, Suzhou, 215100 Jiangsu People’s Republic of China; 3 Department of Pharmacology, Soochow University, Suzhou, 215123 Jiangsu People’s Republic of China; 4 Guangdong Institute of Cardiovascular Diseases & Guangdong Key Lab for Protein Modifications and Degradation, The Second Affiliated Hospital & School of Basic Medicine, Guangzhou Medical University , Guangzhou, 511436 People’s Republic of China

**Keywords:** Acevaltrate, Valerian, Myeloma, Otub1, c-maf

## Abstract

**Background:**

The oncogenic transcript factor c-Maf is stabilized by the deubiquitinase Otub1 and promotes myeloma cell proliferation and confers to chemoresistance. Inhibition of the Otub1/c-Maf axis is a promising therapeutic target, but there are no inhibitors reported on this specific axis.

**Methods:**

A luciferase assay was applied to screen potential inhibitors of Otub1/c-Maf. Annexin V staining/flow cytometry was applied to evaluate cell apoptosis. Immunoprecipitation was applied to examine protein ubiquitination and interaction. Xenograft models in nude mice were used to evaluate anti-myeloma activity of AVT.

**Results:**

Acevaltrate (AVT), isolated from Valeriana glechomifolia, was identified based on a bioactive screen against the Otub1/c-Maf/luciferase system. AVT disrupts the interaction of Otub1/c-Maf thus inhibiting Otub1 activity and leading to c-Maf polyubiquitination and subsequent degradation in proteasomes. Consistently, AVT inhibits c-Maf transcriptional activity and downregulates the expression of its target genes key for myeloma growth and survival. Moreover, AVT displays potent anti-myeloma activity by triggering myeloma cell apoptosis in vitro and impairing myeloma xenograft growth in vivo but presents no marked toxicity.

**Conclusions:**

The natural product AVT inhibits the Otub1/c-Maf axis and displays potent anti-myeloma activity. Given its great safety and efficacy, AVT could be further developed for MM treatment.

**Video Abstract**

## Background

*Valeriana* is a genus of flowering plants in the family Caprifoliaceae and it contains many species including *V. officinalis* L. *s.l., V. wallichii* DC. (*V. jatamansi* Jones), and *V. edulis* Nutt. ex Torr & Gray ssp. *procera* (H.B.K.) F. G. Meyer (*V. mexicana* DC.). These plants can be found around the world, from Northern America, Europe, to many countries of Asia. Extracts from valerian roots are traditionally used as a herbal remedy for many neurological disorders, including insomnia, anxiety, stress, depression, attention deficit disorder, chronic fatigue syndrome, tremors, epilepsy [1, 2]. In addition, valerians are also found with many other pharmacological activities, including blood pressure lowering activity, anti-inflammatory activity, hepatoprotective activity, anticholinesterase activity, antimicrobial activity, and others [2]. However, the anti-cancer activities of valerians are largely unknown.

The valarian roots are the major bioactive parts from which many chemical ingredients have been identified including both water soluble fractions and oil extracts. These active ingredients could be classified as valepotriates, flavones, lignans, sesquiterpenoids, sesquiterpenoids, terpinoids, and others [2], of which the most studied one is valepotriates. Valepotriates are a class of iridoid triesters, which could be divided into the monoene-type (e.g. didrovaltrate and isovaleroxyhydroxydidrovaltrate), and the diene-type (e.g. valtrate, isovaltrate and acevaltrate) [3]. Currently, the mixed compounds or total extracts from dried valarian roots other than the isolated valepotriates are frequently studied. Twenty years ago, a group from Leiden University of The Netherlands found that diene-type valepotriates are active against lung cancer cell proliferation in culture [3]. However, no studies have been reported on specific valepotriates for hematological cancers.

Multiple myeloma (MM) is an incurable malignancy of plasma cells. The transcription factor c-Maf is involved in myelomagenesis including promoting MM cell proliferation, adherence to bone marrow stromal cells, invasion and metastasis [4, 5]. A recent study also found that c-Maf confers to chemoresistance in MM [6]. Consistently, downregulation of c-Maf by genetic inhibition leads to MM cell apoptosis [7] and enhances MM cell sensitivity to the proteasomal inhibitor bortezomib [6]. Therefore, c-Maf could be a therapeutic target of MM.

Recent studies have found that c-Maf protein is degraded via the ubiquitin-proteasomal pathway [8]. c-Maf is ubiquitinated under the direction of the ubiquitin-conjugating enzyme UBE2O [9] and the ubiquitin ligase HERC4 [10]. Moreover, c-Maf ubiquitination could be dynamically regulated by its deubiquitinases such as Otub1 [11]. Otub1 is an OTU family protease that can prevent c-Maf from polyubiquitination thus stabilizing c-Maf and promotes its oncogenic transcription factor activity. Targeting the Otub1/c-Maf could be an ideal target of anti-MM drug discovery. In the present study, we established a drug screen system in HEK293T cells by overexpressing Otub1, c-Maf and luciferase driven by MARE (a Maf responsive element) [11] and further used this system to screen potential natural products against MM. Acevaltrate, one of several potential natural products identified from the screen, displays great activity in suppressing Otub1 and c-Maf thus showing potent anti-MM activity in vitro and in vivo.

## Methods

### Cells and cell culture

Human embryonic kidney cells (HEK293T) were grown in Dulbecco’s modified Eagle’s medium (DMEM). MM cell lines (RPMI-8226, LP1, KMS11, OPM2, U266) were cultured in Iscove's Modified Dulbecco's Media. All the media were supplemented with 10% fetal bovine serum (ExCell Bio, Inc., Shanghai, China), appropriate glutamine, and antibiotics. Primary bone marrow species from healthy donors and MM patients were collected from the Department of Hematology of the First Affiliated Hospital of Soochow University. The study on primary bone marrow cells was approved by the Review Board of Soochow University. Informed consent was obtained in accordance with the Declaration of Helsinki.

### Plasmids and antibodies

The c-Maf and Otub1 plasmids were subcloned into a pcDNA3.1 vector carrying an HA, or Myc tag. The luciferase reporter driven by c-Maf recognition element (MARE) (5′-TGCGAGTGAGGCA-3′) (pGL4-MARE.Luci) was synthesized by GeneWiz, Inc. (Suzhou, Jiangsu, China) [7]. The Otub1-siRNA and control-siRNA were purchased from Ribobio Inc. (Guangzhou, China) [11].

The antibodies used for Western blot were as follows: anti-Otub1 and anti-Ub were from Santa Cruz Biotechnology Co. Ltd (Santa Cruz, CA); anti-glyceraldehyde-3-phosphatedehydrogenase (GAPDH), anti-integrin beta 7 (ITGB7) and anti-c-Maf were from Proteintech Group, Inc. (Wuhan, China); anti-HA and anti-Myc were obtained from MBL Biotech Co., (Beijing, China); anti-poly(ADP-ribose) polymerase (PARP), anti-Caspase 3 and anti-cyclin D2 (CCND2) were purchased from Cell Signaling Technology (Danvers, MA). Antibodies of Otud4, Otud5, and Otud7b were provided by Beyotime Institute of Biotechnology (Haimen, China).

### Screening of the Otub1/c-Maf axis inhibitors

**Fig. 1:**
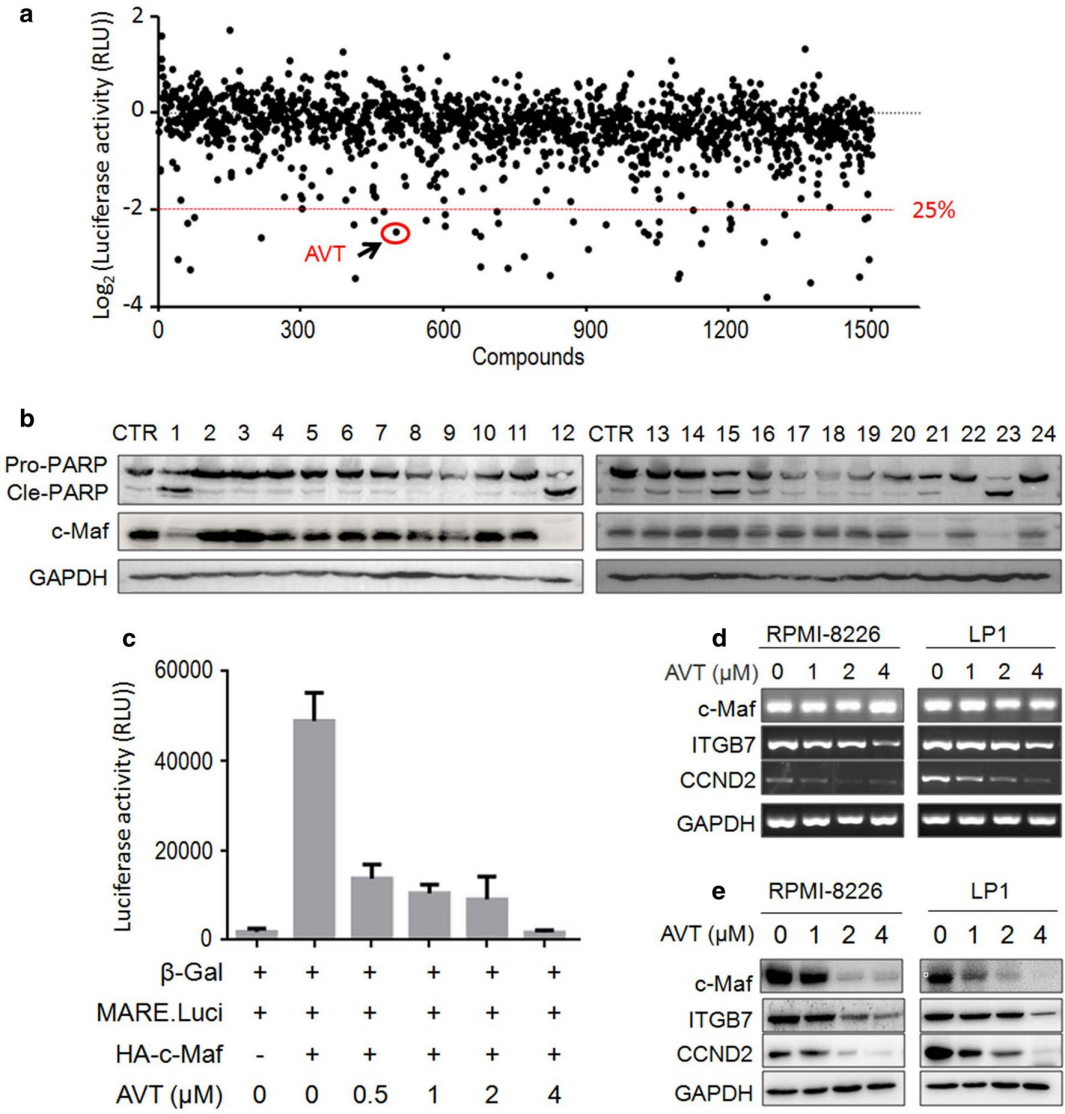
AVT inhibits c-Maf transcriptional activity. **a** HEK293T cells expressing c-Maf/Otub1/MARE.Luci were treated with natural products from TargetMol® or vehicle for 24 h, followed by luciferase assay. **b** RPMI-8226 cells were treated with various candidates for 24 h, followed by cell lysate preparations and WB assays.CTR, vehicle control; 1, flubendazole; 12, AVT; 21, vincristine sulfate; 23, Ouabain. **c** RPMI-8226 cells were transfected with plasmids as indicated for 48 h. Cells were then re-plated and treated with 0.1% DMSO or AVT for 24 h before being harvested for luciferase activity measurements. d-e, MM cells were treated with AVT for 24 h, followed by WB (**d**) or RT-PCR (**e**) assays to evaluate the expression of specific genes

The screen system was established as described previously [11]. Specifically, HEK293T cells were transfected with Otub1, c-Maf and MARE.Luci plasmids for 24 h. The cells were then split into 96-well plates. On the next day, cells were treated with 5 µM of each compound from TargetMol® Natural Product Library (Target Molecule Corp, Wellesley Hills, MA) for another 24 h. Cell lysates were then subjected to luciferase activity assay [11]. The candidates including AVT with activities to inhibit more than 75% were considered for further studies (Fig. 1a).

### Western blot analysis (WB)

Whole cell lysates were prepared from cells of interest as described previously [10]. Cell lysates were then clarified by centrifugation at 10,000×*g* for 30 min and the protein concentrations in the supernatant were determined with a BCA protein assay kit (Beyotime). Equal amount of proteins was subjected to be fractionated by SDS polyacrylamidegel electrophoresis followed by transfer to polyvinylidenedifluoride membranes. The blots were incubated with specific antibodies, followed by incubation with secondary horseradish peroxidase (HRP)-conjugated goat anti-mouseor anti-rabbit IgG (Beyotime) and detection was performed by using the Enhanced Chemical Luminescence reagent and methodology (Beyotime).

### Reverse transcription-PCR

Total RNA was extracted using Trizol® Reagent (Invitrogen). RT-PCR was performed as described previously [7] using a reaction kit containing M-MLV reverse transcriptase (Roche) and ProTaq® DNA polymerase (Roche).The primers used for c-Maf, CCND2, ITGB7 and GAPDH were described previously [7]. The thermal cycle conditions used were as follows: 1 cycle at 94 °C for 5 min; 35 cycles at 94 °C for 30 s; 55 °C for 30 s; 72 °C for 30 s; and 1 cycle at 72 °C for 10 min.

### Lentiviral Otub1 construction

Primers for the complete Otub1 cDNA fragment were as follows: 5′- CCGCTCGAGATGGCGGCGGAGGAACCTCAG -3′ (Forward) and 5′- CGCGGATCCTCTTTGTAGAGGATATCGTA -3′ (Reverse). The Otub1 cDNA fragment was inserted into the pLVX-AcGFP vector (Clontech) within the XhoI and BamHI sites. The viral particles were prepared with a standard method according to the manufacturer’s instructions and included control and package plasmids (Shanghai GeneChem Co., Ltd.). The plasmids were co-transfected into HEK293T cells with the calcium precipitate method. Viruses were obtained 72 h later from transfected cells and were applied to infect the appropriate cells.

### Cycloheximide chase assay

After treated with AVT for 4 h, HEK293T cells were treated with CHX (100 μg/ml, Sigma–Aldrich) for indicated periods as needed. Cell lysates were then prepared for SDS-PAGE and immunoblotting analyses with specific antibodies.

### Cell viability and apoptosis assay

MM cells were plated into a 96-well plate with a density of 5 × 10^4^ cells per well, followed by the treatment of AVT for 24 h. Cell growth and viability were assessed with the MTT assay as described previously [9].To determine cell apoptosis, MM cells treated with AVT were stained with Annexin V-fluorescein isothiocyanate (Annexin V-FITC) and propidium iodide (PI) according to the manufacturer’s instruction (MultiSciences Biotech Co., Ltd, Hangzhou, China). Stained cells were analyzed on a flow cytometer (FACSCalibur®, Becton Dickinson). Apoptosis was measured by flow cytometry as described previously [9].

### Colony forming assay

To evaluate clonogenic growth of bone marrow cells from primary MM patients and healthy donors, cells (6.25 × 10^5^/mL) treated with AVT or buffer control for 24 h. After treatment, cells were washed and equal volumes were plated in triplicate in standard MethoCult® GF H4434 medium (StemCell® Technologies, Vancouver, BC) containing 1% methylcellulose in IMDM, 30% FCS, 1% bovine serum albumin, 3 U/mL of recombinant human erythropoietin, 10^–4^ M of 2-mercaptoethanol, 2 mM of L-glutamine, 50 ng/mL of recombinant human stem cell factor, 10 ng/mL of GM-CSF, and 10 ng/mL of rh IL-3. The number of colonies containing 20 or more cells was counted for statistical analysis.

### MM xenograft assays

The human MM cell lines LP1 and RPMI-8226 were *s.c.* injected at a density of 1 × 10^7^ cells per site into female BALB/c nude mice (5–6 weeks old, Shanghai Slac Laboratory Animal Co. Ltd, Shanghai, China).When tumors were measurable, mice were randomly assigned into two groups. One group received vehicle as the control, and the other one was orally administered AVT at a dosage of 50 mg/kg body weight once daily for 15 days. Body weight and tumor volumes were monitored every day (tumor volume =  (tumor length × width^2^)/2). At the end of the experiment, tumors and blood samples were collected for further studies. This study was conducted according to the protocols of the Soochow University Committee on Animal Care and with the approval of the Ethics Review Board of Soochow University.

### Statistical analyses

Statistical differences between the control and the experimental groups were analyzed by Student’s *t*-test. All statistical tests were two-sided, and a *P* value less than 0.05 was considered statistically significant.

## Results

### Identification of AVT by targeting the Otub1/c-Maf transcription system

Our recent study demonstrated that Otub1 stabilizes c-Maf by preventing its polyubiquitination and enhances c-Maf transcriptional activity [11]. To identify inhibitors of the Otub1/c-Maf axis, a bioactive screen system in HEK293T cells was established by expressing Otub1, c-Maf and c-Maf-driven luciferase (MARE.Luci). These cells were incubated with individual natural products obtained from TargetMol®. The luciferase assay revealed that some compounds could suppress c-Maf activity more than 75% in terms of MARE.Luci activity (Fig. 1a). Several compounds were chosen for further validation by measuring their effects on PARP cleavage and c-Maf downregulation. As shown in Fig. 1b, flubendazole, AVT, vincristine sulfate and Ouabaine displayed potent activity to decrease c-Maf and induce PARP cleavage. Because flubendazole and vincristine sulfate have been reported in MM and Ouabaine is relatively toxic, AVT, a diene-type valepotriate was taken for further studies. To confirm this finding, HEK293T cells expressing endogenous Otub1 were co-transfected with c-Maf and MARE.Luci plasmids, followed by AVT treatment. The result showed that AVT suppressed c-Maf transcriptional activity in a concentration-dependent manner (Fig. 1c). Subsequently, we evaluated the effects of AVT on c-Maf transcriptional activity in MM cells. RPMI-8226 and LP1, two typical MM cell lines, were treated with AVT for 24 h followed by WB and RT-PCR assays. As shown in Fig. 1d and e, AVT decreased the expression of c-Maf protein but not its mRNA, suggesting that AVT modulated c-Maf protein stability. Furthermore, the expression levels of CCND2 and ITGB7, two representative genes modulated by c-Maf, were downregulated by AVT at both mRNA and protein levels (Fig. 1d–e), indicating that AVT downregulated the transcription of these two genes, as expected. Therefore, these assays demonstrated that AVT suppressed c-Maf transcriptional activity by promoting c-Maf protein turnover.

### AVT promotes c-Maf degradation in proteasomes

**Fig. 2:**
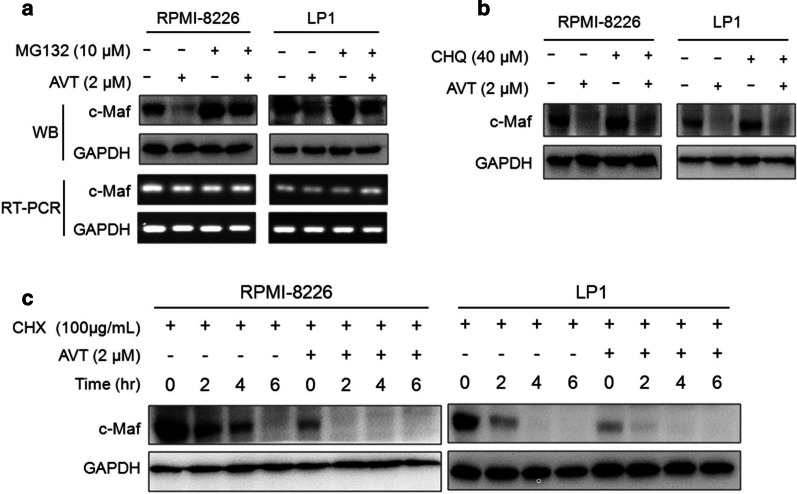
AVT induces c-Maf degradation via the ubiquitin-proteasomal pathway. **a**–**b** RPMI-8226 and LP1 cells were incubated with AVT for 4 h, followed by the treatment of the proteasomal inhibitor MG132 (**a**) or the lysosomal inhibitor chloroquine (CHQ) (**b**). The cell lysates were subjected to WB assays. **c** RPMI-8226 and LP1 cells were treated with AVT 4 h followed by CHX treatment for indicated periods. All cell lysates were subjected to WB assays as indicated

The study above showed that AVT downregulated c-Maf protein in the presence of Otub1. Meanwhile, c-Maf could be degraded via both lysosomal and proteasomal pathways [8] and Otub1 is also associated with the autophagy-lysosome pathway [12] and c-Maf deubiquitination [11]. To find out which pathway is important for AVT-induced c-Maf degradation, MM cell lines were treated with AVT along with MG132 (a proteasomal inhibitor) or chloroquine (a typical lysosomal inhibitor), followed by WB assays. The results showed that c-Maf protein was decreased by AVT, and it was rescued by MG132 (Fig. 2a) but not by chloroquine (Fig. 2b), suggesting AVT-induced c-Maf degradation via the proteasomal pathway. Further, we measured c-Maf degradation by CHX chase assay in the presence of AVT. The results showed that c-Maf was markedly decreased by AVT. In the presence of CHX, AVT significantly reduced the half-life of c-Maf (Fig. 2c). Therefore, AVT induces c-Maf degradation via the ubiquitin-proteasomal pathway.

### AVT interferes with the interaction between Otub1 and c-Maf to increase c-Maf polyubiquitination

**Fig. 3:**
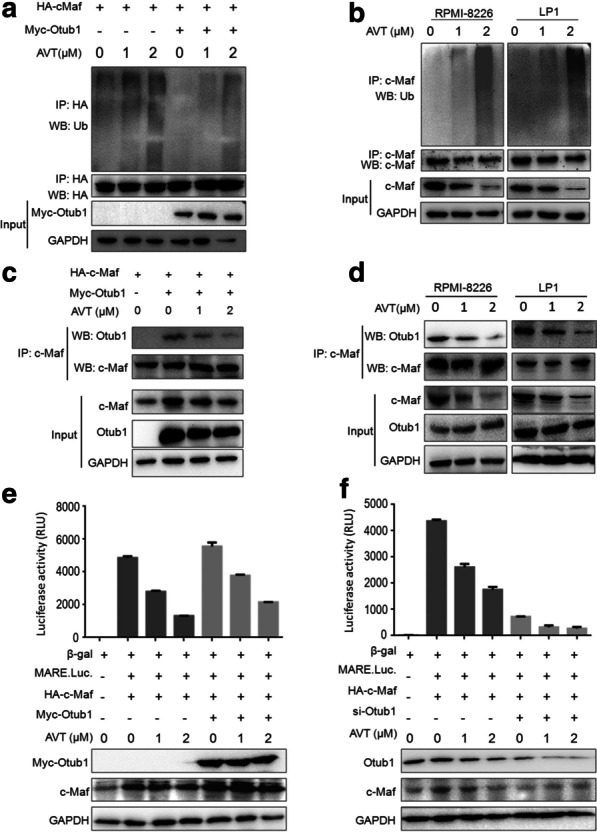
AVT accumulates c-Maf polyubiquitination by disrupting the interaction between Otub1 and c-Maf. **a** c-Maf and Otub1 plasmids were co-transfected into HEK293T cells for 12 h followed by AVT treatment for 24 h. Cell lysates were subjected to a co-IP/WB assay. **b** RPMI-8226 and LP1 cells were treated by AVT for 24 h before being lysed and subjected to co-IP/WB assays. **c** c-Maf and Otub1 plasmids were co-transfected into HEK293T cells for 12 h, followed by AVT treatment. Cell lysates were subjected to a co-IP/WB assay. **d** RPMI-8226 and LP1 cells were treated with AVT for 24 h before being subjected to co-IP/WB assays. **e**–**f**. c-Maf and pMARE.Luci plasmids were co-transfected into HEK293T cells with or without Otub1 (**e**) or Otub1 siRNA (**f**), followed by AVT treatment. Cell lysates were subjected to luciferase and WB assays

AVT was identified from the Otub1/c-Maf system, while Otub1 interacts with c-Maf and prevents its polyubiquitination as its deubiquitinase, therefore, we wondered whether AVT can inhibit Otub1 activity toward c-Maf ubiquitination. To this end, we first evaluated c-Maf ubiquitination in HEK293T cells that expressed Otub1, c-Maf and Ub, and further treated with AVT. As shown in Fig. 3a, the IP/WB assay showed that AVT markedly increased c-Maf polyubiquitination in a concentration-dependent manner but overexpression of Otub1 partly ablated this ubiquitination. And this effect was confirmed in MM cells. As shown in Fig. 3b, c-Maf ubiquitination in both RPMI-8226 and LP1 cells was increased in a concentration-manner by AVT (Fig. 3b). Because Otub1 interacts with c-Maf thus preventing its ubiquitination, we next wondered whether AVT interfered with this interaction between Otub1 and c-Maf. As expected, Otub1 was markedly reduced from c-Maf interacting complex (Fig. 3c) and this action of AVT on Otub1/c-Maf was also confirmed in MM cells (Fig. 3d). Therefore, AVT inhibits the deubiquitinating action of Otub1 on c-Maf by disrupting their interaction. Consistent with this finding, overexpression of Otub1 increased c-Maf transcriptional activity, but it was decreased by AVT in a dose-dependent manner (Fig. 3e). In contrast, when Otub1 was knocked down by its specific siRNA, AVT displayed less activity in terms of c-Maf transcriptional activity in the presence of siOtub1, suggesting that AVT activity might dependent on Otub1 expression. In other words, Otub1 is essential for AVT to inhibit c-Maf activity.

### AVT induces MM cell apoptosis dependent on c-Maf expression

**Fig. 4:**
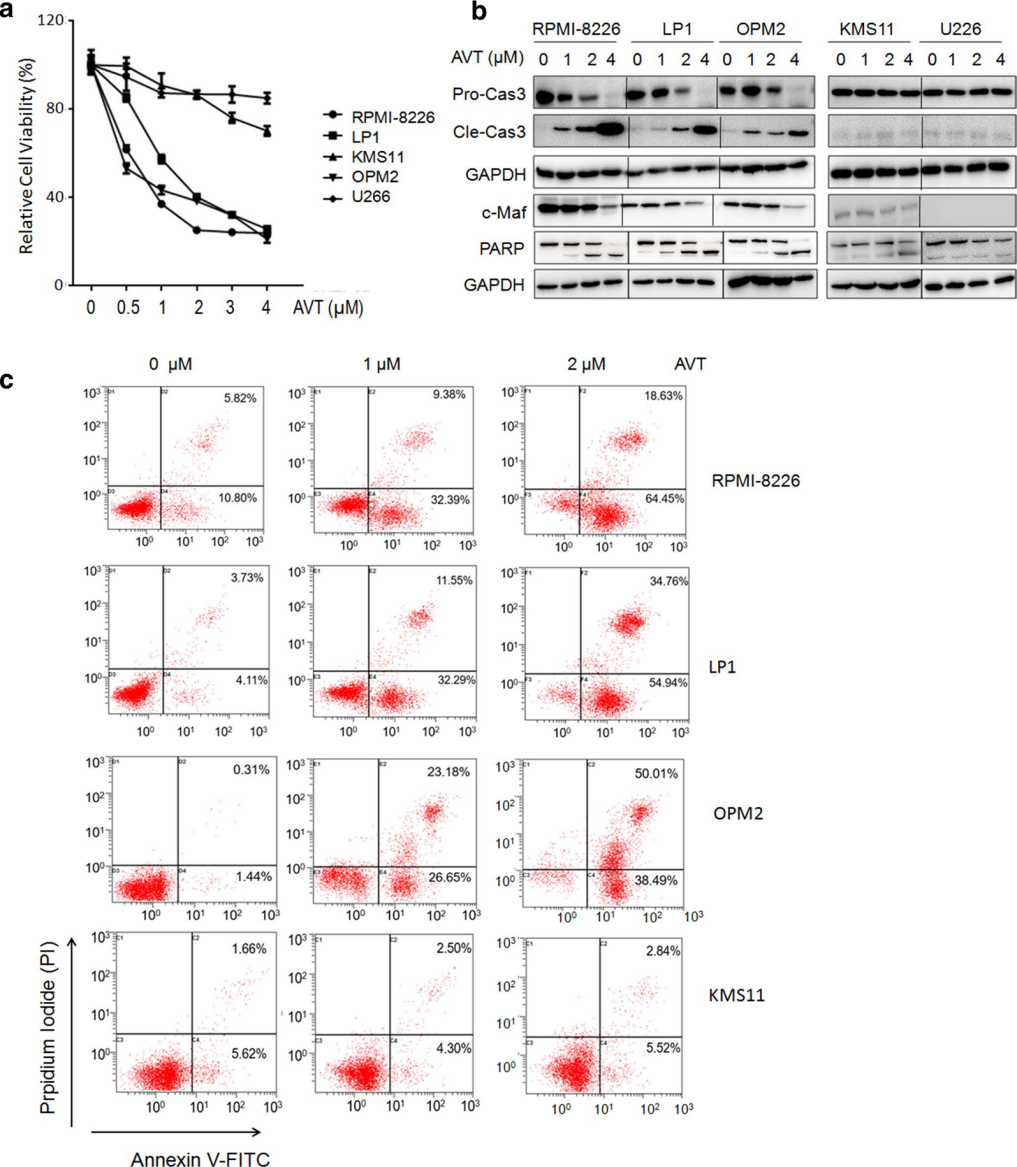
AVT induces myeloma cell apoptosis in the context of c-Maf expression. **a**, A panel of MM cell lines were treated with AVT for 24 h, followed by MTT assay. **b** MM cell lines were treated with AVT for 24 h, followed by WB assay against specific antibodies. **c** MM cell lines were treated with AVT for 24 h, followed by Annexin V/PI staining and flow cytometric analyses

Considering Otub1 and c-Maf promote MM cell proliferation and survival while inhibition of Otub1 and c-Maf results in MM cell apoptosis [7, 11], we therefore wondered whether AVT could induce MM cell apoptosis. To this end, a panel of MM cells were treated with AVT followed by cell viability assay. As shown in Fig. 4a, AVT decreased viability of c-Maf-expressing MM cell lines RPMI-8226, LP1 and OPM2, but not KMS11 and U266 cells that express low or no c-Maf [6]. Subsequently, these MM cells were treated various concentrations of AVT, followed by WB assays against apoptotic hallmark proteins cleaved caspase-3 and PARP. As shown in Fig. 4b, AVT induced cleavage of caspase-3 and PARP, along with c-Maf downregulation in RPMI-8226, LP1 and OPM2 but not in KMS11 and U266 cells. To further evaluate MM cell apoptosis, these AVT-treated cells were subjected to Annexin V/PI staining and flow cytometric analysis. The result showed that AVT strikingly increased the Annexin V positive fraction in all examined cell lines but KMS11 (Fig. 4c), consistent with the above viability and WB assays. Therefore, c-Maf expression could be a determinant factor in AVT-induced MM cell apoptosis. In other words, AVT might prefer to induce apoptosis of MM cells with a high expression of c-Maf.

### Otub1 is critical for AVT to induce MM cell death and c-Maf polyubiquitination

**Fig. 5:**
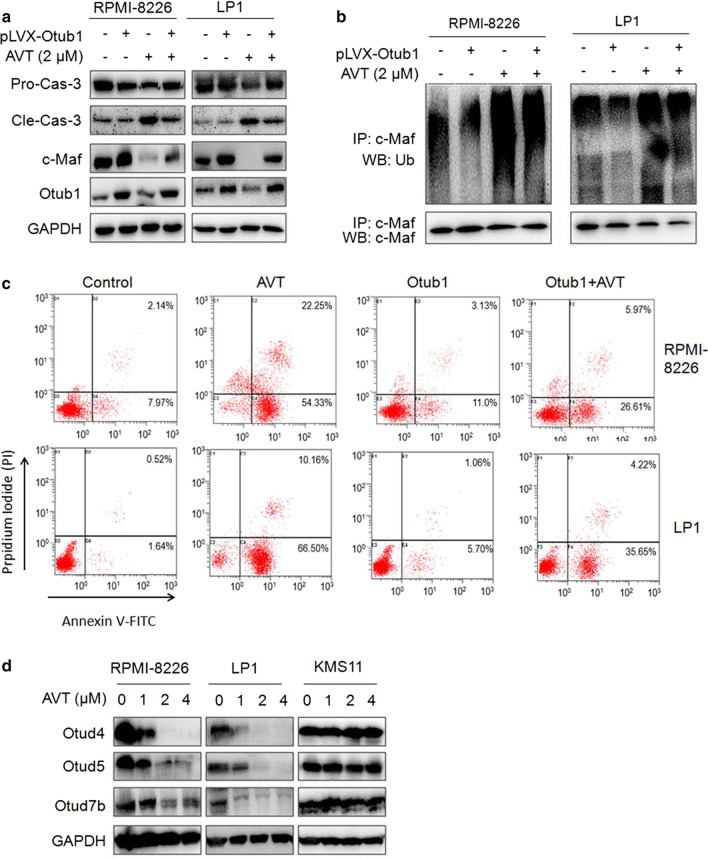
Otub1 expression partly abolishes AVT-induced MM cell apoptosis. **a** MM cells were infected with lentiviral Otub1 for 96 h, followed by AVT treated for 24 h. Cell lysates were then prepared for WB assays against indicated proteins. **b** The cell lysates from a were subjected to co-IP/WB as indicated. **c** RPMI-8226 and LP1 cells were treated as a before being stained with Annexin V-FITC/PI staining and flow cytometric analyses. **d** MM cells were treated with increased AVT for 24 h, followed by WB assays against indicated proteins

The above study has demonstrated that AVT could disrupt the interaction between Otub1 and c-Maf therefore inducing c-Maf degradation in proteasomes and MM cell apoptosis. To convince the role of Otub1 in AVT anti-MM activity, RPMI-8226 and LP1 cells were infected with lentiviral Otub1, followed by AVT treatment. The result showed that AVT drastically reduced the protein level of pro-caspase-3 in both RPMI-8226 and LP1 cells, but it was abolished partly by lentiviral Otub1 (Fig. 5a), suggesting Otub1 expression rescued the apoptosis induced by AVT. To confirm this hypothesis, MM cells after the above treatment and lentiviral Otub1 infection were subjected to IP/WB assays against c-Maf polyubiquitination. As shown in Fig. 5b, Otub1 markedly reduced c-Maf polyubiquitination level but it was reversed by AVT, suggesting AVT inhibited Otub1 activity thus increasing the polyubiquitination level of c-Maf. AVT-induced MM cell apoptosis was probably in association with its inhibition on Otub1. Furthermore, we evaluated cell apoptosis after infection of lentiviral Otub1 and AVT treatment. The Annexin V/PI staining and flow cytometric analysis revealed that AVT-induced MM cell apoptosis was partly ablated by overexpression of Otub1 via lentivirus infection (Fig. 5c).

It is known that Otub1 belongs to the large family of OTU-domain containing Dubs. To find out whether AVT also affected other Otub1 family members, MM cells treated with AVT were subjected to WB against Otud4, Otud5, and Otud7b. As shown in Fig. 5d, AVT elicited potent activity to downregulate all these three Dubs in RPMI-8226 and LP1 but not in KMS11, suggesting that AVT might also target other Dubs in addition to Otub1, however, whether these Dubs are associated with c-Maf should be further studied.

### Acevaltrate impairs MM xenograft growth in vivo

**Fig. 6:**
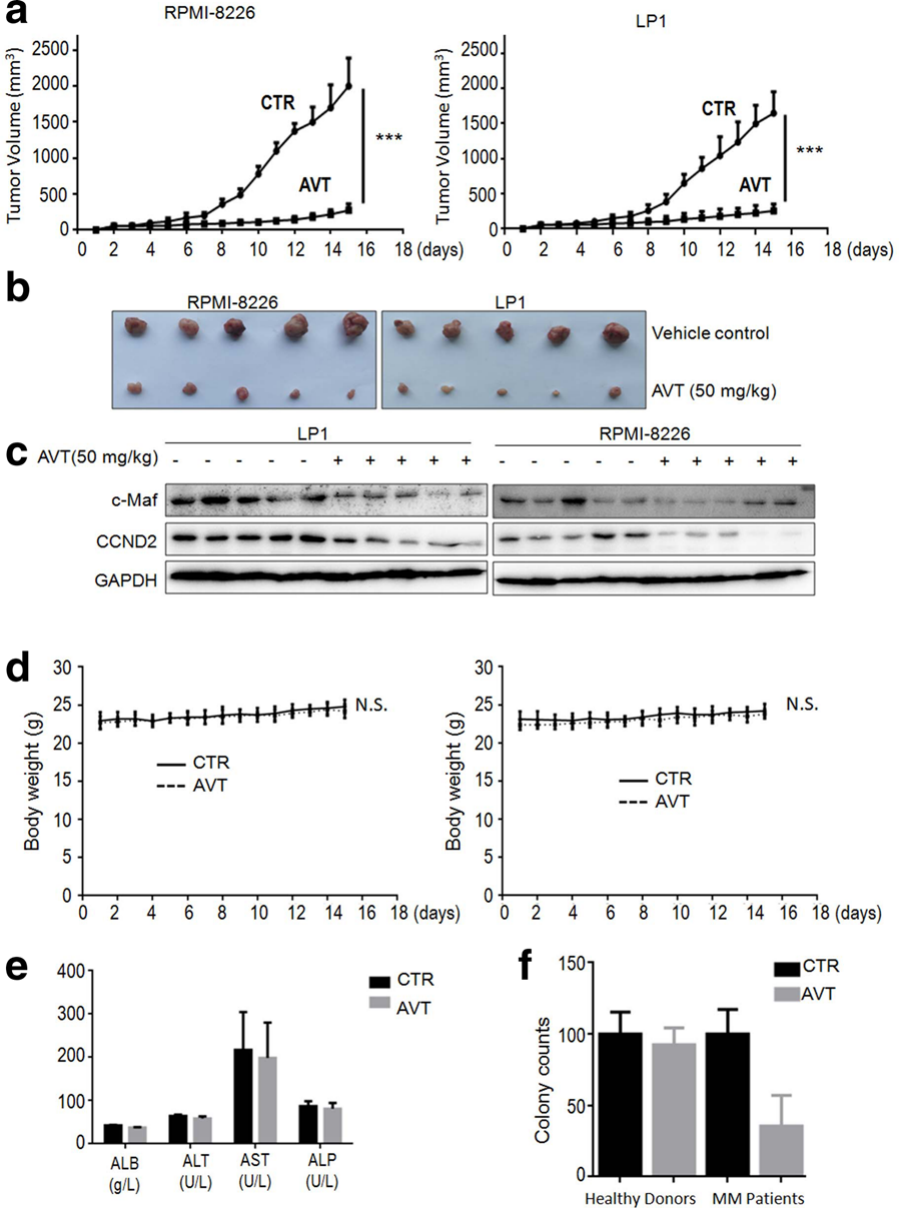
AVT delays myeloma tumor growth in immunodeficiency mice. Two myeloma xenograft models were established by injection of RPMI-8226 or LP1 cells into immunodeficiency mice. **a** Tumor volume growth was monitored every day during 15 d of treatment. Data were expressed as mean ± standard deviation. ***; *p* < 0.001. **b** At the end of the experiment, tumor tissues were dissected. **c** Tumor tissues from each model were subjected to WB analyses for c-Maf, CCND2, and GAPDH with specific antibodies. **d** Mice bodyweight was measured every day during AVT treatment. **e** albumin (ALB), alanine amino transferase (ALT), aspartate amino transferase (AST), and alkaline phosphatase (ALP) from mice blood were analyzed at the end of the experiment. **f** Mononuclear cells from bone marrow collected from healthy donors or MM patients were treated with AVT before being plated in MethoCult® GF H4434 medium to allow colony formation. Colonies were counted on the 14th day of treatment. Colonies with more than 20 cells were counted for analysis. CTR, vehicle control; AVT, acevaltrate

To further clarify whether AVT displays anti-MM activity in vivo, we established two independent MM xenograft models by subcutaneous innoculation of MM cells into nude mice followed by AVT administration for continuous 15 d. The dynamic changes of the tumor sizes and weight indicated that AVT displayed potent anti-MM activity in vivo. As shown in Fig. 6a, AVT strikingly inhibited tumor growth at 50 mg/kg in a time-dependent manner. At the endpoint of the experiment, the tumor sizes were significantly smaller than those in the vehicle control group (Fig. 6b). Moreover, when the tumor tissues were subjected to WB assays, AVT was found to markedly decrease the expression levels of c-Maf and its downstream gene CCND2 (Fig. 6c), suggesting that AVT impaired MM tumor growth associated with c-Maf downregulation, in line with the above studies. However, AVT did not show any overt toxicity in mice in terms of body weight (Fig. 6d) and biochemical analysis of liver function in terms of the values of ALT, AST and ALP (Fig. 6e). Moreover, the colony forming unit assay showed that AVT has no effects on normal bone marrow, but impaired the proliferation and differentiation of blood stem/progenitor cells from MM patients (Fig. 6f). Therefore, AVT impaired myeloma xenograft growth*in vivo* in association with c-Maf degradation.

## Discussion

Otub1 has been reported in many cancer tissues and negatively associated with poor prognosis of cancer patients. At the molecular level, Otub1 stabilizes several oncoproteins such as Snail [13], and FOXM1 [14] as a deubiquitinase that result in cancer cell proliferation and survival. Therefore, inhibition of Otub1 has been proposed as an anti-cancer therapeutic target. In MM cells, Otub1 can stabilize the oncogenic transcription factor c-Maf. Inhibition of Otub1 and c-Maf triggers MM cell death [7, 11]. In the present study, we identified AVT as an inhibitor of the Otub1/c-Maf axis for the potential treatment of MM, a malignant plasma cell disorder.

AVT is isolated from the dried roots of valeriana, a genus of flowering plants most commonly known as valerians. As a member of valepotriates, AVT is active against sleeping disorder and other neurological diseases [15, 16], however, there are very few studies on AVT in its anti-cancer activities. In contrast to valtrate that has been reported with specific activities against breast and ovarian cancers [17, 18], there are only two reports regarding AVT for its anti-oxidant and its cytotoxic activities [3, 19]. Therefore, the present study for the first time identified AVT as a potent active valerian in triggering cancer cell death. As demonstrated in the present study, AVT not only inhibits MM cell proliferation, but also induces MM cell apoptosis and impairs MM growth in vivo, this is the first report that systemically evaluated the anti-cancer effect of AVT.

As stated above, valepotriates have been proposed with multiple bioactivities, but the detailed mechanisms are not yet known. The present study demonstrated that AVT elicits its anti-myeloma activity via inhibiting the Otub1/c-Maf axis therefore activating apoptotic signaling transduction. It is essential for Otub1 to engage with c-Maf before preventing c-Maf polyubiquitination and degradation via the ubiquitin-proteasomal pathway, however, AVT can disrupt this interaction between Otub1 and c-Maf, which further abolishes the deubiquitinating activity of Otub1 towards c-Maf. AVT thus induces c-Maf degradation and suppresses its oncogenic transcriptional activity which is demonstrated by c-Maf modulated genes including CCND2 and ITGB7. CCND2 is a promoter of cell cycle progress from G1 to S phase and contributes to MM cell proliferation [20], while ITGB7 is a member of integrins on cell surface that promotes MM cell adhesion to stromal cells and contributes to MM survival, invasion and metastasis [5]. The present study demonstrated that AVT inhibits MM cell proliferation in culture and impairs MM tumor growth in vivo, which is consistent with c-Maf/CCND2/ITGB7 activity [21]. It is also in agreement with our hypothesis that AVT inhibits the Otub1/c-Maf axis therefore leading to MM cell apoptosis and myeloma regression. This conclusion is also supported by the finding that MM cells such as KMS11 and U266 that express a low level of c-Maf are resistant to AVT but LP1, OPM2 and RPMI-8226 cells that express a high level of c-Maf are sensitive to AVT. However, AVT might also target other proteins and signaling transductions. As shown in Fig. 5d, AVT also decreases Otud4, Otud5 and Otud7b in addition to Otub1/c-Maf. This is probably right. After all, AVT is a natural product that was first identified as a modulator of sleeping disorders by affecting the action of gamma-aminobutyric acid, adenosine, and serotonin neurotransmitters. In terms of cancer treatment, a recent study found that the valepotriate derivatives induce human pancreatic cancer cell apoptosis by inhibiting the PI3K/AKT pathway and activating Noxa signaling [22]. AVT might elicit anti-cancer activities via many other signaling pathways to be identified.

## Conclusions

Natural products are a great source of drug discovery because of their richness in nature and great safety. Various studies at both animal and clinic settings have demonstrated that valepotriates are highly safe [23]. In the present study, we found that AVT mainly acts on the bone marrow stem cells from MM patients but had no effects on those from healthy donors in terms of colony formation assays. Moreover, the animal study should that AVT had no effects on mice body weight and biochemical parameters in terms of liver and kidney function. Given its safety and efficacy in the experimental models of MM, AVT could be further developed as an anti-MM natural product. Targeting at the Otub1/c-Maf axis is a promising strategy for the treatment of MM.

## Data Availability

All data generated or analyzed during this study are included in this published article.

## Abbreviations

AVT: acevaltrate
CCND2: cyclin D2
FITC: fluorescein isothiocyanate
GAPDH: glyceraldehyde-3 -phosphatedehydrogenase
ITGB7: integrin β7
MM: multiple myeloma
HRP: horseradish peroxidase
PARP: poly(ADP-ribose) polymerase
